# Ultrasound-guided double-lumen tube size selection improves intubation success and postoperative airway outcomes in thoracic surgery: a randomized controlled study

**DOI:** 10.3389/fmed.2026.1865696

**Published:** 2026-06-17

**Authors:** Mustafa Duran, Seniyye Ülgen Zengin, Meliha Orhon Ergün

**Affiliations:** Department of Anesthesiology and Reanimation, Marmara University Pendik Training and Research Hospital, Kadikoy, Türkiye

**Keywords:** airway management, double-lumen tube, one-lung ventilation, thoracic surgery, ultrasonography

## Abstract

**Background:**

Appropriate double-lumen tube (DLT) size selection is crucial for safe and effective one-lung ventilation in thoracic surgery. Conventional selection based on sex and height does not account for individual airway variability. This study evaluated whether ultrasound-guided DLT size selection improves intubation success and postoperative airway outcomes.

**Methods:**

In this prospective randomized controlled study, 70 patients undergoing thoracic surgery who required one-lung ventilation were assigned to either a conventional group (DLT size based on sex and height) or an ultrasound group (DLT size based on tracheal diameter measured by ultrasound at the suprasternal notch). The primary outcome was the incidence of postoperative sore throat at 24 h. Secondary outcomes included postoperative cough, first-attempt intubation success, number of attempts, surgeon-rated lung collapse satisfaction, and intraoperative respiratory parameters.

**Results:**

Postoperative sore throat was significantly lower in the ultrasound group compared to the conventional group (8.6% vs. 37.1%, *p* = 0.004). Postoperative cough was also reduced (8.6% vs. 40.0%, *p* = 0.002). First-attempt intubation success was higher in the ultrasound group (97.1% vs. 62.9%, *p* = 0.001). Additionally, peak airway pressure was lower, and dynamic compliance was higher in the ultrasound group (*p* = 0.002 and *p* < 0.001, respectively). Surgeon satisfaction was significantly improved (*p* = 0.005).

**Conclusion:**

Ultrasound-guided DLT size selection improves intubation success, reduces postoperative airway complications, and enhances ventilatory parameters. This approach may provide a simple and effective method for optimizing airway management in thoracic surgery.

## Introduction

One-lung ventilation is an essential component of thoracic anesthesia, and the use of double-lumen tubes (DLTs) remains the most commonly used technique for achieving effective lung isolation (1, 2). Appropriate selection of DLT size is critical for ensuring optimal ventilation and minimizing airway-related complications (3). Oversized tubes may lead to increased airway pressure, mucosal injury, and challenges in tube placement, whereas undersized tubes may result in air leakage, inadequate lung collapse, and compromised surgical exposure (3, 4). Therefore, accurate DLT sizing is a key determinant of both patient safety and surgical success.

Traditionally, anesthesiologists determine the size of DLTs based on simple anthropometric parameters such as sex and height (5). Although this approach is practical and widely used in daily clinical practice, it does not account for individual variability in tracheobronchial anatomy (4). Previous studies have demonstrated considerable interindividual differences in airway dimensions even among patients with similar physical characteristics, limiting the predictive accuracy of conventional methods (3, 4). As a result, relying on generalized formulas may lead to inappropriate tube selection and suboptimal clinical outcomes (6).

With the increasing integration of point-of-care ultrasound in anesthesia practice, airway ultrasonography has emerged as a promising, non-invasive tool for airway assessments (7). Ultrasound is widely used for vascular access, regional anesthesia, and lung imaging, and its application in airway evaluation has expanded in recent years (8, 9). Several studies have demonstrated a strong correlation between ultrasound-measured tracheal diameter and airway dimensions obtained from computed tomography or chest radiography, with good reproducibility in adult patients (10). Furthermore, previous studies have suggested that tracheal diameter may serve as a reliable predictor for selecting an appropriate double-lumen tube size, providing a more individualized approach than traditional anthropometric methods (10). These findings indicate that ultrasound-guided airway assessments may improve the accuracy of tube size selection and potentially optimize airway management in thoracic anesthesia.

Despite these potential advantages, the clinical impact of ultrasound-guided DLT size selection on intubation success and postoperative airway complications remains uncertain (11, 12). In particular, evidence from randomized controlled trials evaluating whether ultrasound-based approaches improve procedural success and patient outcomes compared to conventional methods is limited (13). Moreover, data regarding the effects of ultrasound-guided DLT selection on clinically relevant perioperative outcomes, such as postoperative airway morbidity and ventilatory parameters, remain limited. Therefore, the primary aim of this prospective randomized controlled study was to evaluate the effect of ultrasound-guided DLT selection on postoperative sore throat. Secondary aims included the assessment of intubation success, postoperative cough, and intraoperative respiratory parameters.

## Methods

### Study design and ethical approval

This prospective, randomized controlled study was conducted at a tertiary academic center between August 2023 and May 2024. Ethical approval was obtained from the Marmara University Ethics Committee (Approval No: 09.2022.755), and written informed consent was obtained from all participants prior to enrollment.

The trial was registered at ClinicalTrials.gov (Identifier: NCT06457763). The study was conducted in accordance with the Declaration of Helsinki and reported in line with the CONSORT guidelines.

### Participants

Patients aged 18–75 years with American Society of Anesthesiologists (ASA) physical status I–III who were scheduled for elective thoracic surgery requiring one-lung ventilation with a left DLT were included.

The exclusion criteria were as follows:

- History of difficult intubation
- Previous head and neck surgery or radiotherapy
- CORMACK–Lehane grade III–IV laryngoscopic view
- Pre-existing sore throat, hoarseness, or chronic cough

#### Randomization and allocation concealment

Patients were randomly assigned in a 1:1 ratio to either the conventional group (group 1) or the ultrasound group (group 2) using a sealed opaque envelope technique.

Group assignments were prepared in advance and placed in sequentially numbered, sealed, opaque envelopes. For each patient, an envelope was opened immediately before induction of anesthesia by an anesthesiologist not involved in postoperative outcome assessment.

#### Blinding

Due to the nature of the intervention, the anesthesiologist performing the intubation could not be blinded. However, postoperative assessments were performed by an independent anesthesiologist who was blinded to group allocation. In addition, the surgeon evaluating lung collapse quality was blinded to group allocation.

#### Study groups and DLT size selection

##### Conventional group

In the conventional group, left-sided DLT size was selected according to the patient’s sex and height. Female patients ≥160 cm received a 37 Fr DLT, and those <160 cm received a 35 Fr DLT. Male patients ≥170 cm received a 41 Fr DLT, and those <170 cm received a 39 Fr DLT. DLT size selection in the conventional group was based on commonly used anthropometric criteria described in previous studies (3, 5).

##### Ultrasound group

In the ultrasound group, patients were placed in the supine position with slight head extension. The transverse outer tracheal diameter was measured using a high-frequency linear ultrasound probe (Esaote MyLab, Esaote, Italy) positioned approximately 0.5 cm above the suprasternal notch. Each measurement was performed twice, and the average value was recorded.

DLT size was selected according to the measured tracheal diameter, based on previously described correlations between tracheal width and appropriate left DLT size, as reported by Brodsky and Lemmens (5).

#### Anesthetic management and intubation procedure

All intubations were performed by anesthesiologists experienced in thoracic anesthesia with prior experience of more than 50 double-lumen tube placements. No trainee performed the intubations.

All patients received standard monitoring including electrocardiography, non-invasive blood pressure, and pulse oximetry.

Anesthesia was induced with propofol (2 mg/kg), remifentanil (1 μg/kg), and rocuronium (0.6 mg/kg). Following direct laryngoscopy, the left DLT was advanced through the vocal cords, rotated 90° counterclockwise, and advanced until resistance was encountered.

The insertion depth was determined using the formula: 12 + [height (cm)/10]. Tube position was confirmed using fiberoptic bronchoscopy in all patients. Tracheal and bronchial cuff pressures were adjusted using a manometer and maintained within the recommended range throughout the procedure.

Respiratory parameters, including peak airway pressure and dynamic compliance, were recorded during one-lung ventilation, 15 min after the confirmation of correct tube placement. All patients were extubated in the operating room following completion of surgery according to standard extubation criteria.

#### Per-protocol analysis and patient flow

A per-protocol analysis was preferred because predefined airway-related outcomes could not be appropriately evaluated in patients requiring alternative airway management following failed DLT placement.

A total of 74 patients were assessed for eligibility. After exclusion of 4 patients, 70 patients were randomized and included in the study. Four patients were excluded from the final per-protocol analysis: in three patients, left double-lumen tube placement could not be achieved and alternative airway management was required; in one patient, tracheal intubation could not be achieved, and the patient was awakened.

Therefore, 70 patients (35 in each group) were included in the final analysis.

#### Outcome measures

An intubation attempt was defined as insertion of the double-lumen tube through the vocal cords. Additional attempts included repeated laryngoscopy or tube repositioning following unsuccessful placement.

The primary outcome of the study was the incidence of postoperative sore throat at 24 h. Postoperative sore throat was assessed using a 4-point categorical scale (0 = none, 1 = mild, 2 = moderate, 3 = severe). Secondary outcomes included postoperative cough at 24 h, first-attempt success rate of DLT placement, number of attempts required for successful placement, surgeon-rated lung collapse satisfaction (1 = poor, 2 = moderate, and 3 = good), selected DLT size, tracheal diameter, and intraoperative respiratory parameters including peak airway pressure and dynamic compliance. Respiratory parameters were recorded during one-lung ventilation, 15 min after the confirmation of correct tube placement.

### Statistical analysis

The sample size was calculated based on the expected incidence of postoperative sore throat. Assuming a reduction from 40 to 10%, with an alpha error of 0.05 and a power of 80%, at least 31 patients were required in each group. Considering possible dropouts, 35 patients were included per group.

Statistical analysis was performed using SPSS (version 29.0; IBM Corp., Armonk, NY, USA).

Normality of data distribution was assessed using the Kolmogorov–Smirnov test.

Continuous variables were expressed as mean ± standard deviation or median (range), as appropriate. Between-group comparisons were performed using the independent-samples *t*-test or Mann–Whitney *U* test.

Categorical variables were presented as numbers (percentages) and compared using the chi-squared test or Fisher’s exact test, as appropriate.

Effect estimates were reported with corresponding 95% confidence intervals (CI), and a *p*-value of <0.05 was considered statistically significant.

## Results

**Figure 1:**
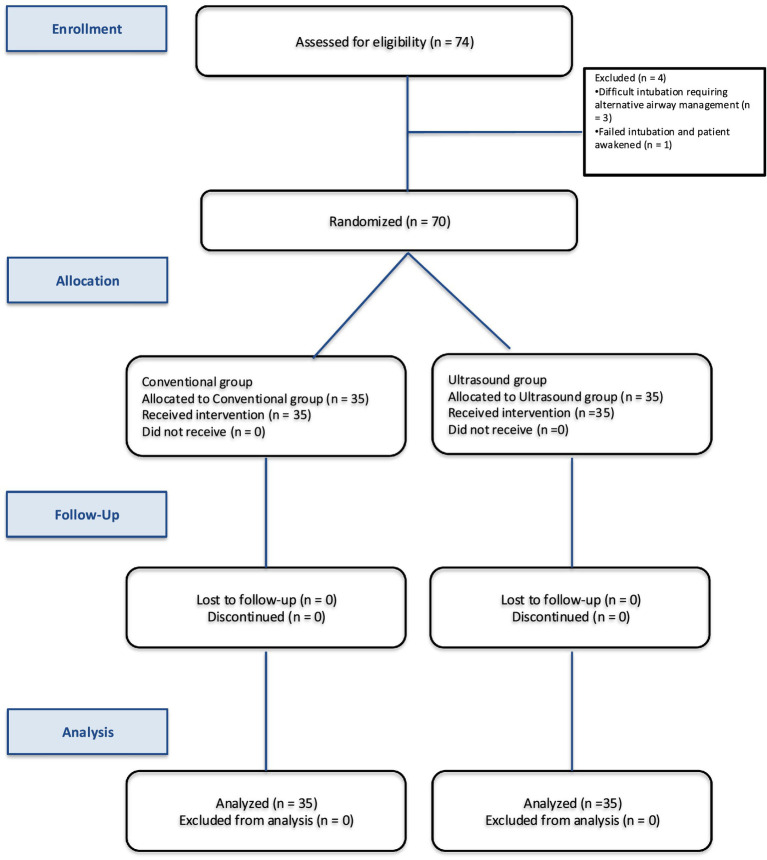
CONSORT flow diagram illustrating patient enrollment, exclusions, randomization, allocation, follow-up, and final analysis.

A total of 74 patients were assessed for eligibility. After exclusion of 4 patients, 70 patients were randomized and included in the study. Four patients were excluded from the per-protocol analysis: in three patients, left double-lumen tube placement could not be achieved and alternative airway management was required, and in one patient tracheal intubation could not be achieved and the patient was awakened. Therefore, 70 patients (35 in each group) were included in the final analysis (Figure 1).

### Baseline characteristics

The demographic characteristics of the patients were comparable between the two groups. The mean age was 57.0 ± 9.6 years in the conventional group and 54.6 ± 10.3 years in the ultrasound group. The conventional group comprised 51.4% of female patients, while the ultrasound group included 37.1% of female patients. Body mass index values were also similar between groups (Table 1). The demographic characteristics of the patients, including age, sex, height, and body mass index, were comparable between the two groups, with no statistically significant differences (Table 1). The duration of surgery was comparable between groups, with no statistically significant difference (Table 1).

**Table 1:** Baseline characteristics of the study population.

	Conventional (*n* = 35)	Ultrasound (*n* = 35)	*p* value
Age, years	57.0 ± 9.6	54.6 ± 10.3	0.316
Sex (female/male), *n*	18/17	13/22	0.229
Height, cm	165.4 ± 7.4	167.3 ± 6.7	0.267
BMI, kg/m^2^	25.8 ± 3.2	25.5 ± 3.1	0.734
Anesthesia duration, min	167.9 ± 14.1	166.5 ± 12.7	0.650

Values are presented as mean ± standard deviation or number. BMI, body mass index.

### DLT size and tracheal diameter

Tracheal diameter measurements and selected DLT size distributions were comparable between groups (Table 2).

**Table 2:** Tracheal measurements and DLT size selection.

Variable	Conventional (*n* = 35)	Ultrasound (*n* = 35)	*p* value
Tracheal diameter, mm	16.86 ± 1.83	16.94 ± 1.83	0.854
35 Fr DLT, *n* (%)	8 (22.9%)	5 (14.3%)	
37 Fr DLT, *n* (%)	10 (28.6%)	10 (28.6%)	
39 Fr DLT, *n* (%)	7 (20.0%)	7 (20.0%)	
41 Fr DLT, *n* (%)	10 (28.6%)	13 (37.1%)	0.781^*^

* Chi-square test compares overall DLT size distribution between groups.

#### Primary outcome

The incidence of postoperative sore throat at 24 h was significantly lower in the ultrasound group compared to the conventional group (8.6% vs. 37.1%, *p* = 0.004) (Table 3).

**Table 3:** Clinical and procedural outcomes.

	Conventional (*n* = 35)	Ultrasound (*n* = 35)	*p* value
Postoperative sore throat, *n* (%)	13 (37.1)	3 (8.6)	0.004
Postoperative cough, *n* (%)	14 (40.0)	3 (8.6)	0.002
First-attempt success, *n* (%)	22 (62.9)	34 (97.1)	0.001
Multiple attempts, *n* (%)	13 (37.1)	1 (2.9)	0.001
Surgeon satisfaction (good), *n* (%)	24 (68.6)	34 (97.1)	0.005
Peak airway pressure, cmH_2_O	25.0 (19–33)	23.0 (18–28)	0.002
Dynamic compliance, mL/cmH_2_O	57.5 ± 8.1	64.7 ± 5.3	<0.001

Values are presented as number (percentage), mean ± standard deviation, or median (range).

This corresponds to an absolute risk reduction of 28.5%, indicating a substantial decrease in postoperative sore throat with ultrasound guidance. The relative risk was approximately 0.23, reflecting a 77% relative reduction. The number needed to treat (NNT) was approximately 4, indicating that the use of ultrasound in four patients would prevent one case of postoperative sore throat (Table 4).

**Table 4:** Effect size analysis of clinical outcomes.

	Absolute risk reduction % (95% CI)	Relative risk (95% CI)	Number needed to treat
Postoperative sore throat	28.5% (10.1–47.1)	0.23 (0.07–0.74)	4
Postoperative cough	31.4% (12.7–50.1)	0.21 (0.07–0.68)	3

Effect size analyses showed clinically relevant reductions in postoperative sore throat and cough, with absolute risk reductions of 28.6 and 31.4%, respectively (Table 3).

#### Secondary outcomes

The incidence of postoperative cough was also significantly lower in the ultrasound group compared to the conventional group (8.6% vs. 40.0%, *p* = 0.002) (Table 3).

**Figure 2:**
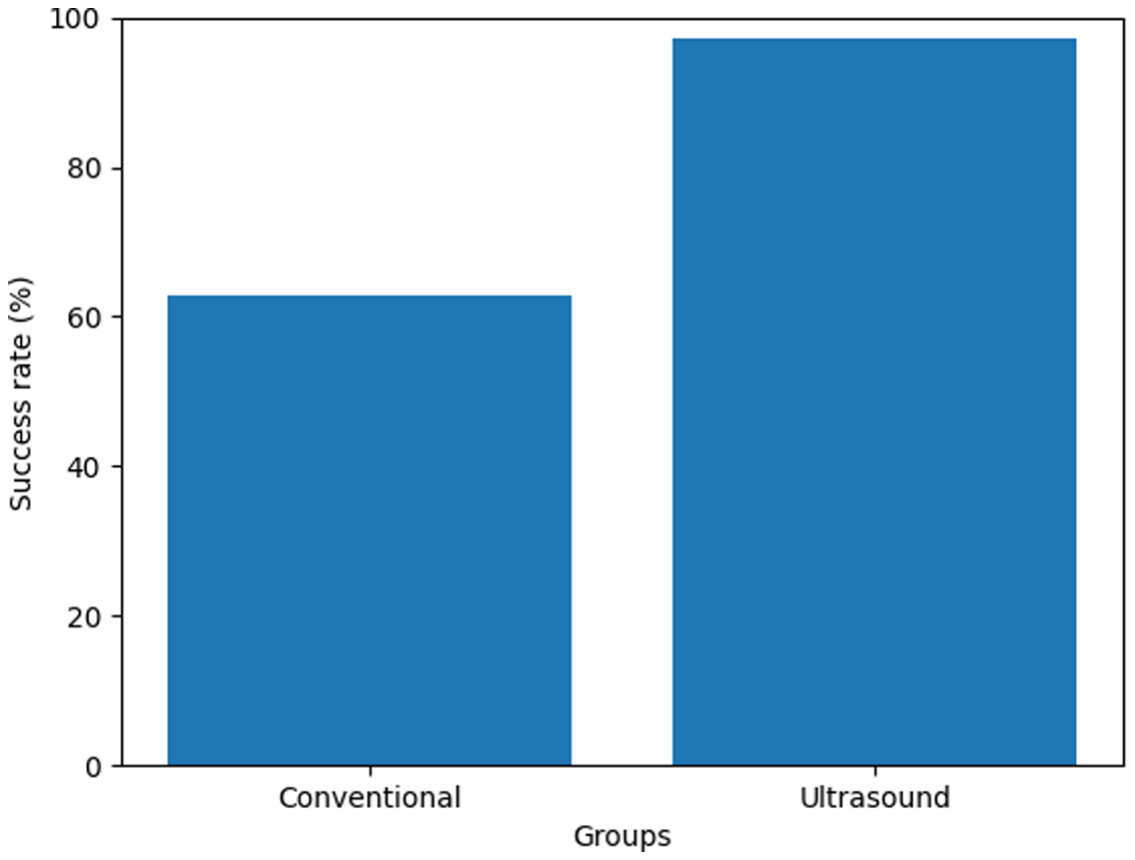
First-attempt success rate of double-lumen tube placement. Ultrasound guidance significantly improved success rate compared with the conventional method (*p* = 0.001).

Similarly, ultrasound guidance significantly improved procedural success, with a higher first-attempt success rate (97.1% vs. 62.9%, *p* = 0.001) (Table 3, Figure 2). Correspondingly, multiple intubation attempts were significantly more frequent in the conventional group compared to the ultrasound group (37.1% vs. 2.9%, *p* = 0.001). No patient required intraoperative replacement with a different DLT size after successful placement.

Surgeon-rated lung collapse satisfaction was significantly higher in the ultrasound group (*p* = 0.005), with a greater proportion of patients classified as having good surgical conditions (Table 3).

### Respiratory parameters

**Figure 3:**
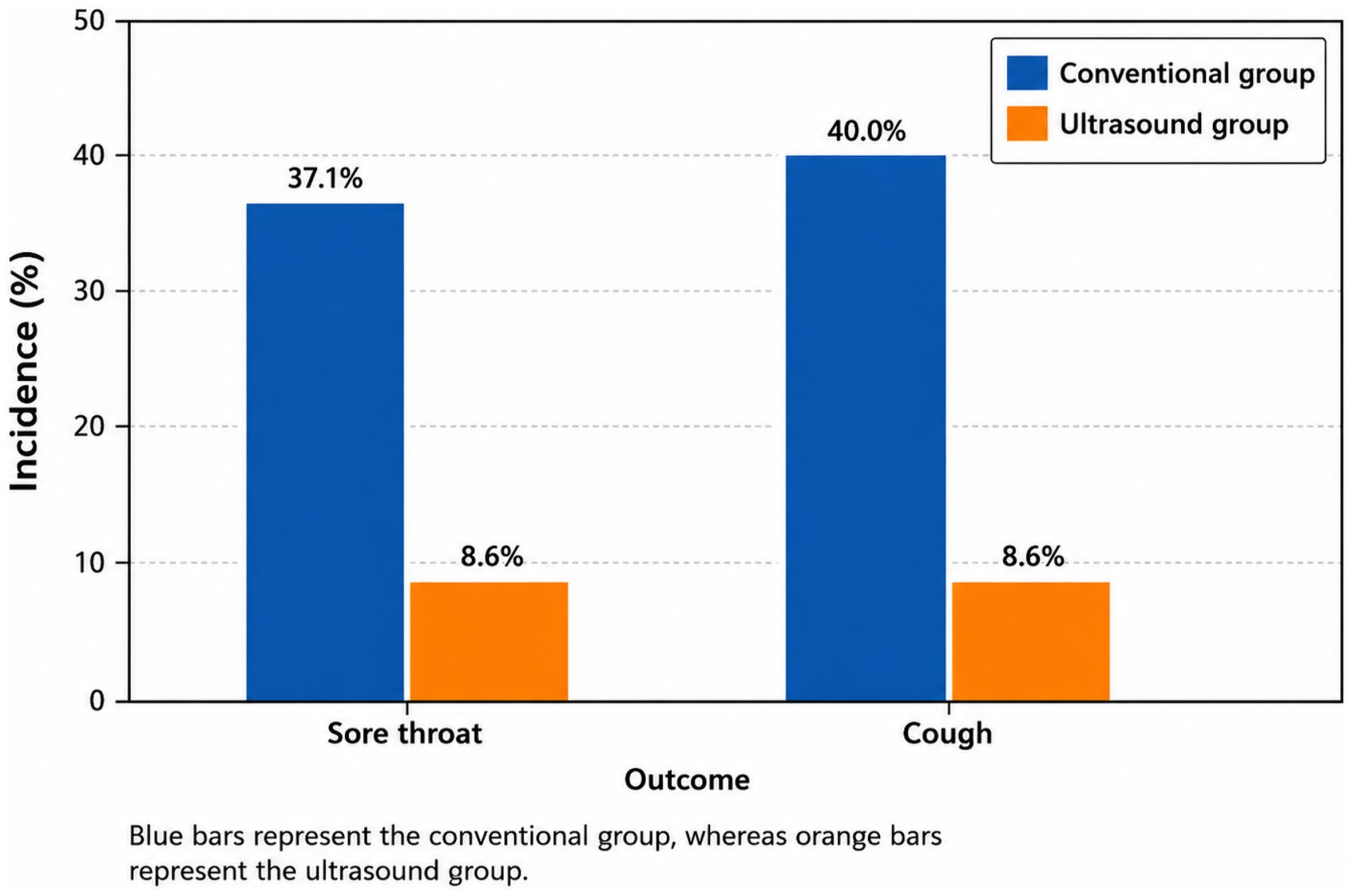
Incidence of postoperative sore throat and postoperative cough in the conventional and ultrasound groups.

Peak airway pressure was significantly lower in the ultrasound group (median 23.0 vs. 25.0 cmH_2_O, *p* = 0.002), while dynamic lung compliance was significantly higher (64.7 ± 5.3 vs. 57.5 ± 8.1 mL/cmH_2_O, *p* < 0.001) (Table 3, Figure 3).

## Discussion

In this prospective randomized controlled study, ultrasound-guided selection of DLT size was associated with a significant reduction in postoperative airway complications and an improvement in intubation success compared to the conventional method (14). Specifically, the incidence of postoperative sore throat and cough was significantly lower in the ultrasound group, while the first-attempt success rate was markedly higher. In addition, ultrasound guidance was associated with more favorable intraoperative respiratory parameters, including lower peak airway pressure and higher dynamic compliance.

Postoperative sore throat is one of the most common complications following airway instrumentation and is influenced by multiple factors, including tube size, cuff pressure, and mucosal trauma (15). In the context of DLT use, airway irritation and trauma may be further increased due to larger tube size and more complex placement (16). Our findings suggest that more accurate tube size selection using ultrasound may reduce airway irritation and trauma, thereby decreasing the incidence of postoperative sore throat. This observation may be explained by improved compatibility between the airway and the selected tube, resulting in less mechanical stress and mucosal injury. Overall, these findings support the concept that individualized airway assessment may contribute to improved postoperative airway outcomes (17). Importantly, no statistically significant difference was observed between groups regarding selected DLT sizes, suggesting that the reduction in postoperative airway complications was unlikely to be solely attributable to smaller tube selection.

Similarly, the reduction in postoperative cough observed in the ultrasound group may reflect improved airway compatibility and reduced mucosal irritation (18, 19). Post-extubation cough is a common phenomenon, frequently resulting from laryngeal and tracheal irritation caused by airway instrumentation (18). Although postoperative cough is often considered a minor complication, it may negatively affect patient comfort and recovery, particularly in thoracic surgery where optimal respiratory function is essential (19, 20). Reduced airway irritation associated with more appropriate tube size selection may therefore contribute to improved postoperative respiratory outcomes.

A key finding of this study is the significant improvement in first-attempt intubation success with ultrasound guidance (21, 22). Accurate estimation of airway dimensions likely facilitates appropriate tube selection, thereby reducing the need for repeated intubation attempts. This is clinically important, as double-lumen tube placement is technically more challenging and associated with a higher risk of airway trauma and complications (21, 23). In addition, repeated or difficult placement attempts may further increase airway injury (23, 24). Improved first-pass success may therefore contribute to safer airway management and better overall outcomes in thoracic anesthesia.

In addition to clinical outcomes, ultrasound guidance was associated with more favorable intraoperative respiratory parameters, including lower peak airway pressure and higher dynamic compliance (25). These findings may indicate a better fit between the airway and the selected tube, resulting in reduced airway resistance and more efficient ventilation. Appropriate tube size selection may therefore play an important role in optimizing ventilatory mechanics during one-lung ventilation (26). Although these parameters are influenced by multiple factors, improved airway-tube compatibility is likely to be a contributing factor.

This study has several limitations that should be acknowledged. First, it was conducted at a single center, which may limit the generalizability of the findings. Second, blinding of the anesthesiologist performing the intubation was not feasible due to the nature of the intervention, which may introduce performance bias. However, postoperative outcome assessments were performed by an independent anesthesiologist blinded to group allocation, which may have reduced assessment bias. Third, some outcomes, such as postoperative airway symptoms, were based on subjective assessments and may be influenced by observer bias. Finally, although the sample size was sufficient to detect differences in primary outcomes, larger multicenter studies are needed to confirm these findings and further evaluate the clinical impact of ultrasound-guided double-lumen tube selection.

In conclusion, ultrasound-guided selection of double-lumen tube size was associated with improved first-attempt intubation success and a reduction in postoperative airway complications compared with conventional methods. These findings suggest that ultrasound may provide a more accurate and patient-specific approach to airway management in thoracic anesthesia. Further large-scale, multicenter randomized studies are warranted to confirm these results and to better define the role of ultrasound in routine clinical practice.

## Data Availability

The datasets presented in this study can be found in online repositories. The names of the repository/repositories and accession number(s) can be found in the article/supplementary material.
